# Assessment of professional identity formation: a transcultural validation of the professional identity essay for brazilian portuguese

**DOI:** 10.1186/s12909-023-04627-0

**Published:** 2023-10-06

**Authors:** Gabrielle Leite Silveira, Verna Monson, Paula Cristina Eiras Poço, Ahmed Haydar, Sigisfredo Luis Brenelli, Fabiana Moreira Passos Succi, Guilherme de Menezes Succi, Milton de Arruda Martins, Patrícia Zen Tempski

**Affiliations:** 1 São Leopoldo Mandic School of Medicine, Campinas - SP, Brazil; 2 Center of Development of Medical Education, School of Medicine of University of São Paulo, Av. Dr. Arnaldo, 455, room 2349, São Paulo, SP 01246-903 Brazil; 3 Kern Institute for the Transformation of Medical Education, Medial College of Wisconsin, Milwaukee, USA

**Keywords:** Medical professionalism, Professionalism, Assessment, Educational strategies

## Abstract

**Introduction:**

Professional identity formation (PIF) is recognized worldwide as an outcome of medical education grounded in the psychology of adult development and the literature on medical professionalism. However, instruments to assess and support PIF are scarce. The Professional Identity Essay (PIE) is an open-ended question assessment of PIF that elicits short narrative responses from learners and that can be analyzed to provide formative feedback and an overall stage of development. In this study, our aim was to translate and adapt the PIE to Brazilian Portuguese.

**Methods:**

We followed a systematic procedure for the translation and cross-cultural adaptation of the instrument. A pilot study was conducted with medical students from the University of São Paulo. After providing individual formative feedback, we administered an online questionnaire to the Brazilian students to better understand the consequences of using the PIE. Content analyses of qualitative data were performed, we employ manifest content analysis, and the categories of analysis emerged from the participants’ speeches.

**Results:**

Students found the instrument’s questions easy to interpret and self-reflective. It also gave students the opportunity to consider their PIF. The PIE was perceived as reliable and brought more awareness of the students’ own processes in addition to a sense of capability to foster their own development. In the same way, the students emphasized the importance of being helped in this process.

**Conclusion:**

We found sufficient evidence of the validity of the PIE in terms of content, face validity, and consequences of use. The PIE enhances self-assurance in PIF through formative assessment and is sensitive to different cultures, making it a potential tool for educators.

**Supplementary Information:**

The online version contains supplementary material available at 10.1186/s12909-023-04627-0.

## Introduction

Professional identity formation (PIF) is at the core of the discussion of the competency-based medical education era [1–4]. Although the formation and values that professional identity relies on vary over time and across cultures [5], the development of professional identity has been acknowledged as a fundamental process in medical education [6]. This identity includes both the way novices transform into full-fledged physicians resulting in “thinking, acting, and feeling like a physician” and the way physicians internalize values, norms, responsibilities, and commitment to patients, society, profession, and themselves [7].

Nurturing professionalism in the curriculum remains a challenge. Cruess et al. [8], while understanding medical education as a community of practice, emphasize characteristics related to hidden curriculum such as tacit knowledge, experimental learning, and role modeling importance in the PIF process. Due to the vast variety of experiences prior to medical graduation, different students are exposed to different effects of the hidden curriculum, which makes PIF heterogeneous among students [9]. Including professionalism in assessments could drive learning and formalize PIF in the curriculum [10, 11].

The Professional Identity Essay (PIE) assessment provides educators or counsellors with a practical approach to measuring PIF, the developmental process of internalizing role concepts, values, and codes of the profession [12]. The PIE was developed for use in professional education, drawing upon the methodology of the Subject-Object Interview (SOI). Research using the PIE or related forms in law, medical, and counseling education demonstrates that the construct produces reliable measurements and that there is validity evidence to support its use as a formative assessment or evaluation tool [12–16].

The PIE is grounded in Robert Kegan’s constructive developmental theory of moral development in adulthood. From his perspective, identity development is a lifelong process and depends on the self-integration of cognitive, social, and emotional processes. This instrument is characterized by oriented short-narrative responses to seven prompts and two invitations to reflect on the meaning of being a member of the profession, the conflicts that the participant will experience, reactions to hypothetical scenarios about failing to meet the expectations of oneself and others, and role models with the goal of bringing the changes that are being processed in participants’ professional identity to consciousness (Table 1).

**Table 1:** The PIE instructions and prompts (modified for use in medicine)

Directions: This essay explores how you understand the meaning of professionalism at this point in your development and how that relates to the formation of an ethical professional identity. Research suggests that the meaning of professionalism and one’s identity with the profession evolves throughout one’s career. Respond as fully as you can to each of the questions. The purpose is to engage you in self-assessment, reflection, and goal setting.	
**Please answer these questions as fully as you can in 1 h.** ***Write at least a paragraph for each question.***	
1. What does being a member of the medical profession mean to you? How did you come to this understanding?	
2. What do you expect of yourself as you work toward becoming a full-fledged physician?	
3. What will the profession expect of you?	
4. What conflicts do you experience or expect to experience between your responsibility to yourself and others—patients, family, and profession? How do you resolve them?	
5. What would be the worst thing for you if you failed to live up to the expectations you have set for yourself?	
6. What would be the worst thing for you if you failed to live up to the expectations of your patients?	
7. What would be the worst thing for you if you failed to live up to what society expects of physicians? How did you come to this understanding?	
8. Think of a physician you consider an exemplar of professionalism. Describe why you chose this person, illustrating with an incident or pattern of decisions or actions that supports your choice.	
9. Reflect on your experiences in medical school or in the community that have been critical in fostering change in your understanding of what it means to be a professional – to be a physician.	

Kalet et al. [19]

The essays are analyzed by trained experts in order to assign an overall stage of Kegan’s theory (adapted for use in professional education), and individual formative feedback is provided. The main aim is to engage students in self-reflection and assessment of their professional identities [14].

As an instrument with open-ended questions, it might be useful for different cultures, and it has already been proven to be useful in different professions [17]. Furthermore, no instrument to assess PIF in our language was found. Thus, our objective was to translate, adapt and validate PIE into Brazilian Portuguese [18].

## Method

### Translation and cultural adaptation of PIE

**Fig. 1:**
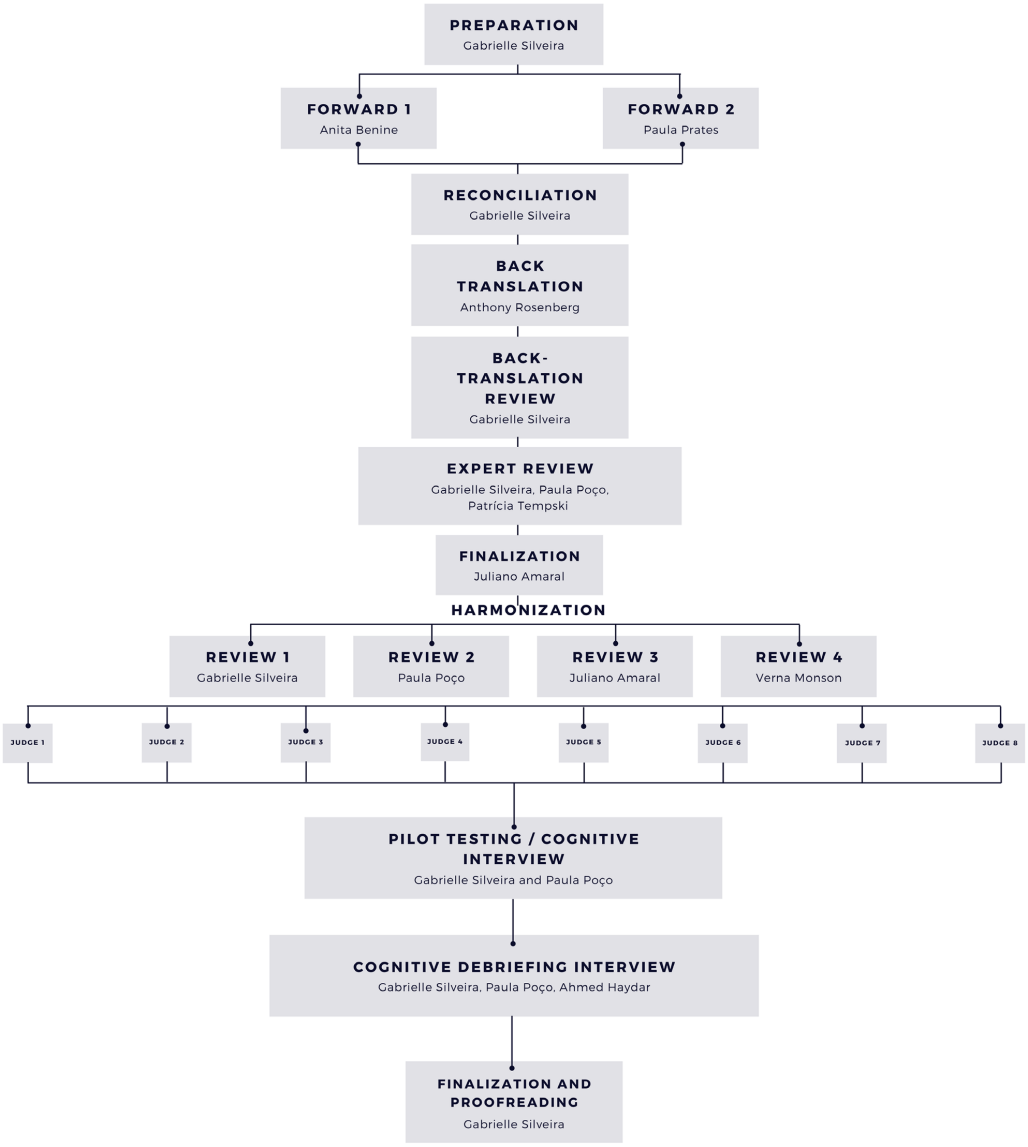
Translation process schema

The steps described below in Fig. 1 were followed in consonance with good practices in the validation and cultural adaptation of instruments [19–21]. A flowchart (Fig. 1) has been designed for a better understanding.

1. The PIE expert (VM) consented to the use of the instrument, and the main researcher (GLS) was trained in the analyses of essays and structuring feedback (classification into Kegan’s PIF stage).
2. Two independent translations were made by professional translators who are native speakers of Brazilian Portuguese.
3. A reconciled version was created based on the synthesis of the two translated versions.
4. A back translation was made by an English native speaker from the reconciled version.
5. In a back-translation review, discrepancies between the original version and the back translation were identified. An expert review was conducted with a psychologist researcher in the field of professional identity (GLS). Two physicians, experts in medical education and with experience in the validation and cross-cultural adaptation of instruments, collaborated during this step. Any doubt about the original version’s expressions was clarified by the instrument expert (VM).
6. A revision of linguistic equivalence was made by a professional linguist, resulting in a pre-final version.
7. The pre-final version was re-evaluated by eight judges (seven medical educators and one medical student) in terms of language equivalence and the content validity of other dimensions. The content validity ratio (CVR) was calculated, and all items with a CVR lower than 0.750 [22] were re-evaluated and modified by PCEP and GLS, which led to the final version.
8. A cognitive interview was conducted with medical students from the University of São Paulo, located in the city of São Paulo, Brazil. We followed the PROMIS method for the interviews [22]. This is an observational study with a convenience sample. The participation was voluntary, and the invitation was made by PCEP to students who have shown in study group some interest in a medical education. The exclusion criterion was not being registered in the medical course and not accepting the invitation. 20 medical students were invited representing the target audience, and nine accepted. They were third- or fourth-year undergraduate medical students (four female and five male aged between 19 and 43).

Special attention was given to items with a lower CVR from the previous step [22]. Students were also invited to respond to the instrument before the cognitive interview, and if no important adjustments were necessary, the same group of students was invited to participate in the pilot study. This step was conducted face to face as all faculty and students had already been vaccinated against COVID-19 and classes were already being held on campus (adequate precautions against COVID-19 infection were respected). Cognitive interviews were recorded for further analysis and will be destroyed once the study is concluded.

### Pilot study

As no important adjustments were necessary after the cognitive interview, the same students were invited to the pilot study. The audio of the session was recorded by GLS for further analysis.

The pilot study was organized in the following steps:

- The instrument was applied by GLS and PCEP. The students completed the PIE within an hour (day one - D1).
- Immediate impacts and feelings were discussed in a small group debriefing session (D1).
- The analyses of essays and written feedback reports were don by GLS.
- Six weeks after D1, students gathered (day two - D2) to individually read each person’s feedback and write portfolio reflections on the following prompts: “Now that you have received your feedback, write your first impressions of your PIE information: – What surprised you? What ‘rings true’? What information is helpful to you?” Two more direct questions were also asked: “What feelings did you experience after receiving formative feedback, and what is the score of the importance of this activity in medical student formation on a rating scale of 0–10 points rating scale?” The last two questions about feelings were an innovation of our research group that was not included in the original instrument.

### Qualitative analysis

Content analysis of the material extracted from the portfolio reflections was conducted by two researchers (GLS and PCEP) following Bardin’s process: pre-analysis; material exploration; treatment, inference and interpretation of results [19]. The researchers independently defined a set of thematic categories and subcategories, that emerged from the participants’ speeches. Different opinions regarding the meaning of specific passages were discussed until a consensus was reached.

### Ethical approval

This study was approved by the University of São Paulo’s ethical committee (Protocol number CAAE:44950921.0.0000.0068). Those who agreed to participate signed a free, prior, and informed consent form after hearing an explanation of the study’s objectives. Access to the main researcher was guaranteed for all the participants.

## Results

In the process of translation and cultural adaptation, during the expert review step, problems were identified with some expressions that needed clarification with VM: “worst thing” and “full-fledged physician”. The author explained the importance of the arousal of feelings during the process and that questions with “worst thing” were used on purposed; therefore, these expressions were maintained in the final version. On the other hand, “full-fledged” had no correspondence in the Brazilian medical profession, and based on the suggestion of VM, it was deleted from the final version. After cognitive interview analysis, only the instrument’s title needed a substantial change so that it would be self-explanatory about its scope.

In the D1 discussion, the group pointed out that the activity was more reflexive than expected and that this kind of activity is opportune because on a daily basis, excess curricular and extracurricular commitments lead to a scarcity of moments of self-contemplation. The following excerpts illustrate this result:

*“ There is a lack of this kind of activity during undergraduate years, moments reserved to think about our origins: ‘Why am I here?’ We need more time to revisit these things.“*

*” It would be very interesting to do this activity in the first year, in the middle and at the end of the undergraduate years to see how expectations will change over time. Things not only get lost, but change…”*.

*“ It was interesting to revisit various parts of my story and see how I have changed.“*

The group also indicated that beyond being self-reflective, the PIE questions were uncomfortable to think about. Thinking about “the worst thing” was directly linked with perceptions of responsibilities associated with becoming a doctor and immediate thoughts about “how to not let bad things happen.” The fact that this kind of assessment is unusual for these students also created a level of discomfort.

“*I think that for a medical school student, who is used to tests and objective assessments, this is not a simple task. It breaks the pattern. It’s uncomfortable to answer a questionnaire for which there is no right or wrong answer*.“

Six weeks after completing the PIE, all students participating on D1 received a formative feedback report from the expert (GLS). The structure of the feedback, following VM’s material, was an explanation of Kegan’s PIF stages, the classification of students’ responses in a specific stage, and new questions intended to foster reflection on one’s own development based on his/her answers. A feedback example is available in supplementary material Appendix 1.

The D2 material was subjected to qualitative analysis that resulted in three categories: potentiality and reliability of the instrument, awareness raising, and help needed in the process of PIF (Table 2).

**Table 2:** Content analysis: themes, subthemes, and example de-identified quotes

Category and subcategory	Exemplar quote
Potentiality and reliability of the instrument	
Potentiality: mobilization, reflection, possible education strategy, optimism in capability to change.	*“I was positively surprised by all the experiences that were included in the process of reflection while writing my answers to the PIE, the reflection at home after this initial process, and now with the feedback. I was surprised at how a few and apparently simple questions can promote such deep analysis.”*
	*“I was very impressed with the depth of feedback and believe that all of the information will be very useful in my process of development (both personal and professional), in particular the comment about responsibility toward the profession, that moved me a lot.”*
	*“The entire process was useful for me. It is very good to have a guided reflection during undergraduate years. I think reflection by myself is already helpful, but the possibility to have feedback about this process is incredible. The questions raised in the feedback gave clarity to thoughts that were already inside me but needed fuel to become stronger.”*
	*“It made me think about things I have never thought about, or even ones that I have thought about but not with dedication, paying attention… At last, this feedback was very profound and filled with relevant information about me, which made me think again about which things I can improve in myself and in my life. This is of great value and brought me a feeling that there is a way, a direction and that it’s possible to be better…”*
Reliability format, veracity, identification	*“During the entire process, what surprised me the most was how much I could identify myself in the questions from the feedback, how much my answers to those questions allowed for an accurate analysis of my personal and professional identity.”*
	*“Since I started undergraduate work, I’ve been doing therapy, and many of the topics raised by GLS were things that took me a while to externalize. Reading this information in my feedback clarified many of these topics for me.”*
Awareness raising awareness of own process; autonomy perception; possible paths	*“It is good to know that the development of professional identity is a dynamic process. This knowledge motivated me to stop ignoring my doubts and difficulties about becoming a physician and face it as a continuous path toward development.”*
	*“I think that mainly the questions asked to clarify my answers, which gave me a possible starting point to extend further, but also the idea that as we move forward, we can learn better how to deal with contradictions that surround us.”*
Help needed in the process self-care	*“I still don’t have the total capacity of looking beyond the negative possibilities in the profession and also maintaining my full tasks for self-care.”*
	*“The comment about self-care was very valuable to make me think about how to make it happen…”*

### Potentiality and reliability of the instrument

Positive reactions were identified for D2 responses, not only for formative feedback. Students reinforced the potentiality of generating profound reflection, which extended beyond the moment of responding to the PIE. This reflection, even if uncomfortable, was perceived as necessary. The students also experienced feelings of identification with the feedback, particularly when faced with their own words. This format is perceived as a powerful strategy.

### Awareness raising

It was possible to identify those students who had increased awareness of their own process of PIF and possible ways to achieve development.

It is good to know that the development of professional identity is a dynamic process. This knowledge motivated me to stop ignoring my doubts and difficulties about becoming a physician and face it as a continuous path toward development.

Similarly, we could identify the perceptions of more autonomy and resilience throughout students with higher levels of PIF, who were able to handle the contradictions and challenges of professional practice. Some students were surprised by their classification in the PIE, so we identified a dissonance between what one is or perceives to be, what one would like to be, and what one actually is. Related to this, some students wrote about the idealizations of their own medical practice.

### Help needed in the process of professional identity formation

The instrument enabled students to realize that they still had to learn about perspective taking and self-care.

I still don’t have the total capacity of looking beyond the negative possibilities in the profession and also maintaining my full tasks for self-care.

**Fig. 2:**
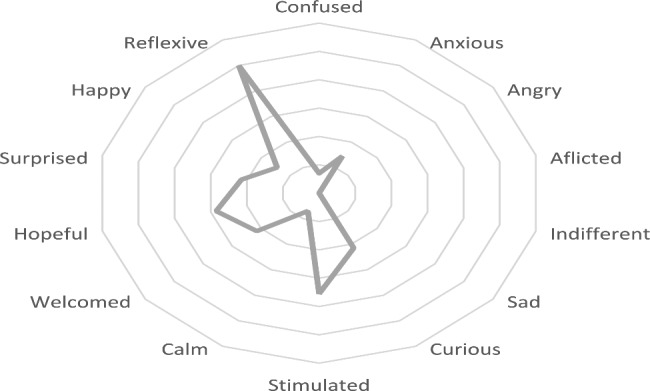
Feelings that arose after the pilot PIE feedback report

After D2, 100% of the students referred to being reflective, 71% said they were feeling more stimulated, and 57% were more hopeful. Figure 2 shows the overall responses for each group (Fig. 2).

When questioned about recommending the activity to fellow students, 70% gave an assessment of 9 or 10 [23], and 30% gave an assessment of 8.

## Discussion

The PIE is an innovative and systematic instrument to assess PIF and provide formative feedback. As such, it is considered a formal strategy to foster professionalism development during under-graduation alongside other important initiatives focusing on real and contextualized scenarios [20] as well as faculty (role model) development.

The PIE feedback reports can be personalized by incorporating the values and norms of different cultures, maintaining the main characteristic of the instrument, which aims to promote reflection and help students to see a possible way forward during PIF. We observed that students welcomed and were intrigued by this educational tool, confirming its potential as a strategy in a competency-based professionalism curriculum [14].

Worldwide research on PIF has already made it clear that this process is based on and promotes student awareness [21, 24, 25]. Thus, students gain a consciousness of the learning and morals of the hidden curriculum. The perception of the disconnect between what is taught and what is actually practiced is important to prevent unprofessional and unempathetic attitudes [26, 27]. This phenomenon deserves special attention in countries where students start medical school immediately after high school graduation such as Brazil and most Latin American countries. They are in late adolescence and early adulthood, stages considered by developmental psychology authors [28–30] to be more vulnerable to external influences.

Organizations such as the General Medical Council (GMC) and the American Board of Internal Medicine’s (ABIM) ‘Project Professionalism’ have suggested that an important component of the development of medical students’ professionalism is self-assessment. Additionally, several educators have suggested that peer evaluation may be a useful complement. Both formats help students develop the ability to make judgments, a necessary skill for academic and professional life. By judging the work or behavior of others, students gain insight into their own performance, an important element of professional competence [31].

In addition, students all over the world are demanding strategies that develop autonomy, self-knowledge, and metacognition [23]. In consonance with the international scenario, a Brazilian study of final-year medical students showed that they also need (and ask for) a curricular activity that offers opportunities to reflect on their PIF [32]. The PIE’s feedback reports have worked as a roadmap and guide for students’ self-development. Furthermore, the participants of this study felt less lonely on the journey and felt more hopeful.

Being more self-assured about their PIF makes it possible for students to come closer to the social contract. When they are confident about their PIF, they develop greater respect for each other, are more empathetic, and act with altruism. In the end, they guide their own development toward being a full-fledged physician and “thinking, acting, and feeling like a physician.”

Aiming for this full development, an instrument to assess PIF that is sensitive to different cultures, such as the PIE, can be fruitful. Furthermore, since teaching and learning professional attitudes are closely linked to cultural and emotional aspects of each individual, the validation of potential instruments that can evaluate and support PIF is an important requirement in Brazil as it is in international medical education.

## Conclusion

We collected sufficient evidence to support the translation, adaptation and validation of PIE to be used with medical students in Brazil. Method proved to be reliable and can be added to information about the feelings of participants after the experience. Its feasibility on a large scale still needs to be assessed.

### Electronic supplementary material

Below is the link to the electronic supplementary material.

Supplementary Material 1

## Data Availability

The datasets analyzed during the current study are available from the corresponding author on request.
