# Supplementary material for: Assessment of professional identity formation: a transcultural validation of the professional identity essay for brazilian portuguese
Source: BMC Med Educ. 2023 Oct 6;23:738. doi: 10.1186/s12909-023-04627-0 (PMC10559411; doi:10.1186/s12909-023-04627-0)
Supplement: Supplementary file 1 — Supplementary Material 1 [file 12909_2023_4627_MOESM1_ESM.docx]

**PROFESSIONAL IDENTITY ESSAY (PIE) ASSESSMENT**

(PILOT VERSON)

**DATE:**

**TO:** ANONYMIZED

**PREPARED BY:** Gabrielle Silveira. MSc.

**REGARDING:** Exploring Your Professional Identity Development

**INTRODUCTION**

The Professional Identity Assessment (PIE) is a measure of professionalism defined as a lifespan developmental construct. This approach stems from an influential report on professionalism in medicine by the Carnegie Foundation recommending that medical education utilize an evidence-based approach to assessment of ethical professional identity. Central to the underlying theory are four ideas:

(1) the development of a professional identity involves increasing complexity of mental processes,

(2) cognitive development runs parallel to social-emotional development,

(3) our level of mental complexity shapes hidden assumptions that filter our views of ourselves and others, as well as our behavior, and

(4) development proceeds from being externally defined to internally or self-defined. This letter will provide you with detailed feedback about the analysis of your PIE responses and suggestions for further self-reflection about your professional identity.

**THE Professional Identity Assessment (PIE)**

The PIE asked you respond to several short essay questions:

(1) What does being a member of the medical profession mean to you? How did you come to this understanding?

(2) What do you expect of yourself as you work towards becoming a full-fledged physician?

(3) What will the profession expect of you?

(4) What conflicts do you experience or expect to experience between your responsibility to yourself and others—patients, family, profession? How do you resolve them?

(5) What would be the worst thing for you if you failed to live up to the expectations you have set for yourself (patients, family, and profession)? How did you come to this understanding?

(6) Think of a physician you consider an exemplar of professionalism. Describe why you chose this person, illustrating with an incident or pattern of decisions or actions that supports your choice.

Questions asked in the PIE are conceptually broad and developed to elicit responses that can be coded along a continuum of professional identity developmental stages. There are no “right or wrong” answers. Your responses were coded by a lifespan developmental educational psychologist who has extensive training and research experience in the methodology, developed by Dr. Robert Kegan of Harvard University. **The intention of this assessment is to provide you with feedback on your assessment and offer some reflective questions intended to foster continued growth in your professional identity. Your results will not be used as a summative assessment of your current performance as a resident physician.**

There are four broad levels of mental complexity that are relevant for professional education. They include:

- ***Independent Operator* (Stage Two)**^[[1]](#footnote-1)^ is characterized by external definitions of self, a predominance of “either-or” thinking, limited perspective taking ability, and an emphasis on the mastery of technical skills. This stage is characteristic of adolescence and early adulthood.
- ***Team-Oriented Idealist* (Stage Three)** is characterized by increased social perspective taking ability among allies or one’s in-group members. Understanding and expectations of the professional role is externalized, shaped by interpersonal relationships, observing others, and following the norms and status quo within organizations without question. Some adolescents and most adults are in this stage.
- ***Self-Defined* (Stage Four)** involves the ability “to step back enough from the social environment to generate a ‘seat of judgment’ or personal authority that evaluates and makes choices about external expectations.” The independence of judgment and problem solving abilities of stage 4 translate to greater fidelity to one’s sense of self within the professional role. At stage 4, one can discern negative social influences that can erode one’s professional identity and integrity. Effectiveness within high-level professional or leadership roles requires stage 4 capacities.
- ***Self-Transformed* (Stage Five)** is characterized by the ability to examine one’s self-authored personal authority, recognize the limits of any one system of constructing meaning, and seek out novel or alternative systems. A recognition of the interdependencies of different systems or ways of being, and an ability to reconcile contradictory or seemingly paradoxical ways of constructing meaning is a hallmark of the emergence of the self-transforming mind. Very few adults reach this stage.

**YOUR RESULTS**

Your results are shown below as a range of content from your PIE assessment that could be coded in one of the stages or transition phases. Your responses suggest you are in **Stage 3 (Socialized Mind)**. The range of content within your response is indicated below by the solid line and brackets.

**Kegan Stages of Mental Complexity**

| ***Externally Defined*** | | ***Self-Defined*** | |
| --- | --- | --- | --- |
| **Independent Operator (Stage 2)** | **Team-Oriented (Stage 3)** | **Self-Defined (Stage 4)** | **Self-Transformed (Stage 5)** |
|  | | | |

### Keep in mind that there is no one stage that is “better” per se, but rather, there are different challenges and advantages associated with each stage. In general, one assumes more independence and authenticity as one’s identity grows towards Stage 4 or Self Definition.

**Analysis of your PIE responses**

In general, your answers reflect a **self-defined professional identity**. Strengths of your narrative were your awareness and your desire to practice the medicine you believe in, even with negative models. This represents a mind of its own making that is capable of looking around it critically, capable of distinguishing between different opinions, formulating its own basis of judgment. The result is a "self-authorship" of your identity, regardless of your environment.

Thank you for sharing your feelings about what-if failures if you didn't meet your or others' expectations. It was a pleasure to read your narrative.

**Here are some selected points for reflection:**

1. In response to question 6, when answering about the patients' expectations, there is the feeling that there may be future suffering if they perceive any failure, injustice, inadequacy or negligence on their part.

**Comment:** How do you think you would understand a setback or failure in a more complex and self-determined way? Reframing failures and setbacks contribute to the development of a more complete and resilient self-defined professional identity. In addition, it is worth thinking about how much, in current medical practice, decision-making should, whenever possible, be shared. This can help you deal with this possible suffering.

2. When answering about your responsibility towards the profession, I highlight the passage in which you said: “It keeps haunting me that I will be technically incapable, that my knowledge and my clinical reasoning are insufficient and that I should know more and even have studied more.” Even knowing that you are a dedicated person, you are not convinced of it.

**Comment:** How will you know if you've done enough? As you experience events that challenge you to question "how much is enough," step back and reflect on how you will know when you've worked hard enough. Reflecting and rethinking your motivations will be important to preserve your health and well-being, so that you can take care of others and yourself.

3. Another important point was related to self-care and care for your family. You talk about frustration, a desire to be able to take care of yourself more, to dedicate yourself more to yourself and your family. Also, fear of overloading yourself at times.

**Comment:** How do you plan self-care, reflective practice, or management of your emotions and thoughts that could indicate when this is happening? Reactions to stress or disappointment that can damage your health or well-being can be reframed as challenges to improving your ability to regulate emotions. Psychotherapeutic approaches can be useful in creating new coping strategies for such situations.

**SUMMARY:** Professional identity integrates thought, emotion, and behavior. With sufficient support, growth in our professional identity occurs as we grapple with challenging experiences that expand our capacities to make sense of problems and our professional role. Scholarship on professionalism points to the importance of self-reflection as part of developing a professional identity defined as a “process involving habits of thinking, feeling, and acting.”

**ADDITIONAL FOLLOW-UP**

If you wish to discuss your results in more detail, please contact me and schedule an appointment:

Thank you for your participation and interest.

Prof. MSc. Gabrielle Leite Silveira

gabrielle.silveira@usp.br

**ACKNOWLEDGEMENTS**

This feedback letter was adapted from a memorandum written by Muriel J. Bebeau, Ph.D. at the University of Minnesota School of Dentistry.

**ADDITIONAL RESOURCES**

Deepening your understanding of developing your professional identity requires your active involvement in the curriculum at the State University of São Paulo, with special attention to curriculum elements on professionalism and medical ethics.

You also have the opportunity to participate in extracurricular activities held at your university.

Below is a bibliography of excellent journal articles and book chapters that explore the topic of professional identity development.

**BIBLIOGRAPHY**

- Bebeau MJ & Faber-Langendoen K. Remediating Lapses in professionalism. 2014. In Kalet A & Chou CL (eds). Remediation in medical education: a mid- course correction. New York. Springer. 103-127
- Blank, L, et al. Medical professionalism in the new millennium: a physician charter 15 months later. Annals of Internal Medicine 138.10 (2003): 839-841.
- Brosnan CP and Bourdieu P. The theory of medical education: thinking “rationally” about medical students and medical curricula. In Brosnan C and Turner BS (eds) Handbook of the sociology of medical education. 2009. London, UK. Routledge. P. 51-69
- Burford B. Group processes in medical education: learning from social identity theory. Med Ed. 2012: 46
- Cooke M, Irby DM, and O’Brien BC. Educating physicians: a call for reform of medical school and residency. Jossey-Bass. San Francisco, Ca. 2010
- Cruess RL, Cruess SR, Boudreau JD, Snell L & Steinert Y. Reframing medical education to support professional identity formation. Acad Med 2014; 89: 1446- 1451
- Cruess RL, Cruess SR, Boudreau JD, Snell L & Steinert Y. A schematic representation of professional identity formation and socialization: a guide for medical educators. Acad Med 2015; 89 in press
- Frost H and Regehr G. “I AM a Doctor”: Negotiating the Discourses of Standardization and Diversity in Professional Identity Construction. Acad Med 2013, 88(10): 1-8
- Goldie J. The formation professional identity in medical students: considerations for educators. Med Teach 2012:34: e641-e648
- Hafferty FW. Professionalism and the socialization of medical students. In Cruess RL, Cruess SR, and Steinert Y (eds) “Teaching Medical Professionalism”. Cambridge Univ Press. 2009. p53-73
- Hilton SR and Slotnick HB Proto-professionalism: how professionalization occurs across the continuum of medical education. Med Educ 2005; 39: 58-65
- Horlick M, Masterton D, and Kalet A. Learning skills of professionalism: a student-led professionalism curriculum. Med Educ 11 (2006): 1-8.
- Jarvis-Selinger S, Pratt DD, and Regehr G. Competency is not enough: integrating identity formation into the medical education discourse. Acad Med 2012; 87: 1185-1191
- Kalet AL, et al. Promoting professionalism through an online professional development portfolio: successes, joys, and frustrations. Acad Med 82.11 (2007): 1065-1072.
- Kenny NP, Mann KV and MacLeod, H. Role modeling in physicians' professional formation: Reconsidering an essential but untapped educational strategy. Acad Med, 2003, 78, 1203-1210
- Lempp H. Medical-school culture. In Brosnan C and Turner BS (eds) Handbook of the sociology of medical education. 2009. London, UK. Routledge. P. 69-71
- MacLeod A. Caring, competence and professional identities in medical education. Adv in Health Sci Educ. 2011; 16: 375-394
- Mann K. Learning and teaching in professional character development. In Kenny N and Shelton W (eds) Lost virtue: professional, character development in medical education. London, UK. Elsevier. 2006 (Advances in Bioethics, volume 10), 145-185
- Mann K, Gordon J and MacLeod A. Reflection and reflective practice in health professions education: a systematic review. Adv in Health Sci Educ. 2008; 595- 621
- Merton RK, Reader LG, and Kendall PL (eds). The student physician: introductory studies in the sociology of medical education. Cambridge MA: Harvard Univ Press. 1957
- Monrouxe L. Identity, identification and medical education: why should we care? Med Educ 2010; 44:40
- Monrouxe L, Rees CE, & Hu W. Differences in medical students’ explicit discourses of professionalism: acting, representing, becoming. Med Educ 2011; 45: 585-602
- Monrouxe LV. Identities, self and medical education. In Walsh K (ed). Oxford Textbook of Medical Education.Oxford UK. 2013, p 113-123
- Rockfeld J, Horlick M, and Kalet A. "Setting our own standards: a student‐led professionalism curriculum for preclerkship students." Med Educ 37.5 (2003): 483-483.

1. We omit Stage One, as this is typically relevant only to early childhood. [↑](#footnote-ref-1)
